# Leaf and Root-Associated Fungal Assemblages Do Not Follow Similar Elevational Diversity Patterns

**DOI:** 10.1371/journal.pone.0100668

**Published:** 2014-06-27

**Authors:** Aurore Coince, Tristan Cordier, Juliette Lengellé, Emmanuel Defossez, Corinne Vacher, Cécile Robin, Marc Buée, Benoît Marçais

**Affiliations:** 1 INRA, UMR 1136 INRA Université de Lorraine «Interactions Arbres-Microorganismes», Labex ARBRE, FR EFABA, Champenoux, France; 2 INRA, BIOGECO, UMR1202, Cestas, France; 3 Univ. Bordeaux, BIOGECO, UMR1202, Talence, France; 4 Irstea, UR EMGR Ecosystèmes Montagnards, 38402 St-Martin-d'Heres, France; Institute for Plant Protection (IPP), CNR, Italy

## Abstract

The diversity of fungi along environmental gradients has been little explored in contrast to plants and animals. Consequently, environmental factors influencing the composition of fungal assemblages are poorly understood. The aim of this study was to determine whether the diversity and composition of leaf and root-associated fungal assemblages vary with elevation and to investigate potential explanatory variables. High-throughput sequencing of the Internal Transcribed Spacer 1 region was used to explore fungal assemblages along three elevation gradients, located in French mountainous regions. Beech forest was selected as a study system to minimise the host effect. The variation in species richness and specific composition was investigated for ascomycetes and basidiomycetes assemblages with a particular focus on root-associated ectomycorrhizal fungi. The richness of fungal communities associated with leaves or roots did not significantly relate to any of the tested environmental drivers, *i.e.* elevation, mean temperature, precipitation or edaphic variables such as soil pH or the ratio carbon∶nitrogen. Nevertheless, the ascomycete species richness peaked at mid-temperature, illustrating a mid-domain effect model. We found that leaf and root-associated fungal assemblages did not follow similar patterns of composition with elevation. While the composition of the leaf-associated fungal assemblage correlated primarily with the mean annual temperature, the composition of root-associated fungal assemblage was explained equally by soil pH and by temperature. The ectomycorrhizal composition was also related to these variables. Our results therefore suggest that above and below-ground fungal assemblages are not controlled by the same main environmental variables. This may be due to the larger amplitude of climatic variables in the tree foliage compared to the soil environment.

## Introduction

Forest microbial assemblages hold major roles in ecosystem functioning. However, the distribution patterns of fungal assemblages are poorly understood because few studies have been performed at large geographical scales [1]–[2]. The climatic factors influencing the microbial richness and composition are equally still poorly understood compared to macroorganisms. The diversity of macroorganisms decreases with increased latitude [3] and, depending on the group, a hump-shaped distribution or a decrease in species richness with elevation is observed for plants, vertebrates and invertebrates [4]–[5]. These large-scale distribution patterns are of special interest in the context of climate change. Indeed, there is an increasing amount of evidence showing changes in plant communities subject to global warming, in particular along elevation gradients with a shift in the distribution of plants species notably more pronounced at higher elevations [6]–[7].

While it appears that there is a considerable fungal diversity, communities of fungi have been less studied compared with those of macroorganisms. This is due to the difficulty in describing microbial communities adequately. However, advances in molecular techniques, such as the recent high-throughput sequence-based technologies now allow far easier characterisation of fungal assemblages and improved estimation of fungal species richness. Today, sequence-based identification is recognized as a powerful method that has significantly improved our perception of fungi in a variety of environmental conditions and habitats [8]–[10], particularly along environmental gradients [11]–[13].

Microorganisms might not follow the elevational diversity patterns generally observed for macroorganisms. Recent findings suggest that bacteria diversity may not decrease with elevation [14]–[15], and may be higher at mid-elevation (850 m) [18]. Recent studies have shown no change of fungal richness to be associated with elevation [17]–[18]. However, a majority of studies have conclusively noted a decrease in ectomycorrhizal (EcM) diversity associated with elevation [12], [19]–[21]. In addition to the diversity, the composition of fungal assemblages varies with elevation, as reported for fungal assemblages of the beech phyllosphere [11], mycorrhizal fungi [12], [19], [22] and fungal wood decomposers [17]. Climatic variables may explain part of these variations in diversity or composition of the fungal assemblages. Indeed, the mean annual temperature and precipitation explained the observed patterns of EcM fungal richness and assemblage structure along elevation and latitudinal gradients [10], [12], [16]. However, other factors may drive these patterns, particularly in the case of EcM fungi [10], [16], [23]. Noteworthy, the host plant and the soil pH could be the major drivers of below-ground fungal community diversity and composition [12], [16], [19], [24], [25].

In this study, we aimed to determine whether the richness of root-associated and leaf fungal assemblage matches the elevation diversity gradient observed for the majority of organisms *i.e.* shows a decrease with elevation. We hypothesized that i) fungal species richness decreases with higher elevation and lower temperature and ii) leaf-associated fungal assemblages are more constrained by climatic variables than root-associated assemblages because the below-ground assemblage does not experience environmental changes as large as the above-ground assemblage does.

For this purpose, richness and composition of the fungal assemblages were analyzed along elevation gradients in three different mountainous regions of France. We focused on beech dominated forests to alleviate a potential host effect. The leaf “or phyllosphere” fungi we studied were all species inhabiting both the surface and the interior of the leaves [9], the root-associated fungi were those associated with fine roots.

## Materials and Methods

### Ethics Statement

Field sampling was permitted by the Office National des Forêts and the private owner of the St Nicolas forest site. No protected species was sampled.

### Site description and sampling design

Three gradients were located in the Vosges, the Alps and the Pyrenees mountains in France. Forest stands were chosen at three different elevations in the Vosges and the Alps and at five elevations in the Pyrenees. We selected stands with mature and dominant (>50% of the basal area) beech trees (*Fagus sylvatica* L.).

For each elevation site, three plots of five contiguous beech trees were selected. The trees were approximately 5 meters apart within a plot with distances between each plot of approximately 25 meters. Three leaves per tree, from different branches, were sampled between the 29^th^ June and the 9^th^ July 2010 as described in [11]. At the same time, three soil cores were sampled per tree (approximately 50 cm^3^ with 10 cm depth) at 1 m from the trunk in the north, south-west and south-east directions.

The leaves were placed in individual plastic bags containing 10 ml of silica gel each (Sigma-Aldrich, St. Louis, MO, USA) to ensure that they completely dried within a few hours. The plastic bags were stored at 16°C before DNA extraction. Four discs per leaf were cut and were ground into a homogenous powder. For a given tree, the three soil cores were pooled and stored at 4°C before processing (maximum 20 days, without visible mould development). The entire root system was sorted from the soil with a 5-mm-mesh sieve and frozen until processing. The root systems were then gently washed in tap water to remove soil particles along with broken root tips that had been washed out. To select for EcM fungi, the thicker roots (over 3 mm diameter) were discarded and the fine-roots (including residual rhizospheric soil) were pooled, lyophilized and ground 10 sec in a ball mill. Soil analyses were carried out by the Laboratoire d'Analyse des Sols d'Arras (http://www5.lille.inra.fr/las) for pH (water method), total C (NF ISO 10694), total N (NF ISO 13878) and P contents [26]. The mean annual temperature and the annual precipitation at each elevation site were both obtained from the AURELHY method (Analysis Using the Relief for Hydrometeorology) for the 1971–2001 period [27] (Table 1).

**Table 1:** Environmental data of the three elevation gradients.

Region	Site name	Elevation	Longitude	Latitude	Mean annual	Annual	Soil	Carbon	Nitrogen	Phosphorus
(Gradient)					temperature	precipitation	pH	content	content	content
		(m)			(°C)	(mm)	(water)	(g/kg)	(g/kg)	(g/kg)
Alps	Vizille	750	E 5°47′	N 45°04′	10.7	965.8	5.4	52.83	3.51	0.21
Alps	Montsec	1100	E 5°48′	N 45°04′	10.1	1001.7	4.3	116.67	7.02	0.32
Alps	Pic de l'Oeilly	1450	E 5°50′	N 45°05′	9.00	1148.3	4.8	76.27	4.46	0.2
Pyrenees	Laveyron	131	W 00°13′	N 43°45′	13.1	918.7	4.6	20.60	1.15	0.05
Pyrenees	Lourdes	488	W 00°05′	N 43°05′	12.0	1504.5	4.8	48.63	3.73	0.25
Pyrenees	Arras-Sireix	833	W 00°08′	N 42°58′	10.2	1306.0	4.3	114.33	5.97	0.27
Pyrenees	Haugarou	1190	W 00°12′	N 43°00′	9.3	1434.0	6.4	98.87	6.19	0.16
Pyrenees	Lienz	1533	E 00°04′	N 42°53′	7.5	1465.1	5.0	60.97	4.47	0.34
Vosges	Lignéville	380	E 5°59′	N 48°07′	9.1	976.3	4.0	53.27	2.75	0.13
Vosges	St Nicolas	550	E 6°56′	N 47°44′	9.0	1485.8	4.5	117.00	6.54	0.25
Vosges	Guebviller	1180	E 7°02′	N 47°55′	7.3	1672.4	4.2	113.33	7.75	0.33

The element contents in soil and the pH were averaged per site for clarity purpose but were measured in the three plots per site.

### DNA isolation and 454-pyrosequencing

The leaf samples were processed as previously described [11]. The total DNA was isolated with a CTAB phenol/chloroform/isoamyl alcohol protocol. Three leaf DNA isolations from the three different leaves were performed per tree. For homogenized root samples, the total genomic DNA was isolated from 50 mg of fine root powder per sample using the DNeasy Plant Mini Kit (Qiagen, Courtaboeuf, France). For each root DNA sample, approximately 15 mg of polyvinylpolypyrrolidone (PVPP) was added to neutralize putative PCR-inhibitors. Manufacturers' instructions were then followed.

The Internal Transcribed Spacer 1 (ITS1) region was targeted as a recognized DNA barcode for fungal identification [28]. The fungal ITS1 region was amplified from the metagenomic DNA with the forward ITS1F (CTTGGTCATTTAGAGGAAGTAA) and reverse ITS2 (GCTGCGTTCTTCATCGATGC) primers. Before amplification, the primers were adjoined by a self-generated multiplex identifier (MID) and the adaptor A (CGTATCGCCTCCCTCGCGCCATCAG) or by only the adaptor B (CTATGCGCCTTGCCAGCCCGCTCAG) for 454 Titanium pyrosequencing of multiplexed amplicons. The adaptor A and the MID were fused with ITS2 for leaf samples and with ITS1F for root samples. Sequencing was unidirectional and started with the A adaptor.

The amplification of the rDNA ITS1 for the trees from the same plot was done with the same tag-encoded primer set. PCR mix preparation was done in 20 µL. For leaf samples, the protocol is described in [11] and is similar to the one followed by root samples. The final concentrations were: 0.4× enzyme buffer, 1.5 mM MgCl2, 1.12 mg/mL bovine serum albumin, 0.2 mM dNTP each, 0.4 mM each tagged primer (ITS1F and ITS2), 0.05 U/µL Taq polymerase (Sigma-Aldrich, St Louis, MO, USA). DNA extracts from root samples were diluted 100 times. The GeneAmp system 9700 was used with 50% ramp to decrease the rate of temperature change during the cycles. The program was as follow: 5 min at 95°C, 35 cycles of 30 sec at 95°C, 40 sec at 53°C and 45 sec at 72°C, and a final step 7 min at 72°C. The PCR reactions were done for each sample separately (11 sites ×3 plots ×5 trees) and amplicons from the same plot were then pooled [29]. Each PCR product was purified with the AMPure XP purification kit (AgenCourt Bioscience, Fullerton, CA, USA) for leaf samples or on a MultiScreen_HTS_ PCR Plate MSNU030 (Millipore Corporation, Billerica, MA, USA) for root samples. The latter procedure consisted of two washings with 100 µL H_2_O and final resuspension in 40 µL H_2_O. The concentration of each sample was measured with the NanoDrop ND-1000 spectrophotometer and the samples were pooled in equal proportion. In total, we obtained independent libraries corresponding to 66 leaf and root tagged samples, sequenced with three independent runs. The 454 pyrosequencing was done at the Génoscope (Evry, France). The raw data were deposited on the Sequence Read Archive website (http://www.ncbi.nlm.nih.gov/sra) under the study accession ERP003510. The three libraries are under the following experiment accessions: ERX280868 (alias TCA.AQN_GOTS_G0YLLAW02) for the root samples, ERX280870 (alias TCA.AQN_FOTS_GYUGVSB04) for the leaf samples from the Pyrenees, and ERX280871 (alias TCA.AQN_HOTS_G2Q8WDE03) for the leaf samples from the Alps and the Vosges.

### Bioinformatics

The sequences were sorted into different files according to their multiplex identifier (MID) using sfffile software, with no mismatches allowed for the MIDs, which were then removed. The sequences were trimmed with trim.flows in Mothur version 1.26.0 with the default parameters [30].

The ITS1 was extracted using Fungal ITS extractor version 2 [31]. After extraction of the ITS1 region, the sequences were filtered based on a minimal length of 100 bp. Out of a total of 370 449 sequences, 228 104 (61.6%), were retained for downstream analyses. The Molecular Operational Taxonomic Units (MOTUs) were generated by clustering the sequences with Uclust version 3.0 (Usearch version 6.0.152) [32]. The similarity threshold was set at 97% as a suggested consensus in the literature [33]. The taxonomic assignation of a consensus sequence generated by Uclust was done against a curated GenBank database (July 2012) selecting for ribosomal sequences of eukaryotes and against unidentified sequences in www.ncbi.nlm.nih.gov using the Basic Local Alignment Search Tool (BLAST) algorithm version 2.2.23 [34], with the filter turned off (*i.e.* segments of the query sequence that have low compositional complexity were not masked off). This setting decreased the number of sequences with no BLAST hits. Ten BLAST hits were considered when available. The phylum name was extracted from the NCBI taxonomy corresponding to the GenInfo identifier (GI number). An additional BLASTN was carried out online for MOTUs with no hit identified. This allowed us to determine the phylum for few MOTUs. For the assignation at the genus level, only BLAST results for which identity >90% and e-value <e^−50^ were considered. The percentage of identity, given in the BLASTN result, was the ratio of the length of identical bases over the length of the alignment. To increase the robustness of the assignation, the MOTUs were assigned to the genus if at least eight blast hits were congruent with each other for the genus name or if all would be congruent when fewer hits were available. This threshold was chosen arbitrarily but allowed us to avoid selecting the best blast hit which may have been misleading. The species name was determined only for MOTUs with a genus assignation and for which the percentage identity of a BLAST hit was equal or greater than the ITS1 variability among phylum. The ITS1 variability among phylum was calculated from the Table S3 provided by Nilsson and collaborators (2008) [35]. The ITS1 variability for each phylum was calculated as the mean ITS1 intra-specific variability within each phylum weighted by the number of sequences (29 502 sequences in all in [35]) and was as follows: Ascomycota, 2.88%; Basidiomycota, 4.98%; Chytridiomycota, 7.81%; Glomeromycota, 9.48%. The mean ITS1 intra-specific variability within the Zygomycota group was 4.13%. Some synonyms were corrected by referencing the website Species Fungorum (http://www.speciesfungorum.org). The MOTUs were assigned to the species level if at least six BLAST hits were congruent with each other for the species name or all should be congruent when fewer hits were available. The threshold was again chosen arbitrarily. The MOTUs taxonomically assigned to a same species or matching to the same GI number at >90% identity and for an e-value <e^−45^, were subsequently grouped together reducing further the total number of MOTUs. Many MOTUs were then discarded according to the following criteria: 1) non-fungal MOTUs; 2) MOTUs supported by only one sequence (singletons) or 3) MOTUs with no hit found in the additional BLASTN and supported by less than 10 sequences. The two last criteria may be indicative of possible sequencing artefacts, and this is why these MOTUs were not used in the analysis.

### Statistical analyses

The leaf and the root-associated assemblages were analysed separately. An assemblage was defined as the addition of all fungal molecular operational taxonomic units (MOTUs, a reasonable proxy for species) living in a particular niche (roots or leaves). Ascomycete and basidiomycete assemblages of both roots and leaves were also analysed separately, as well as a functional group, the root-associated EcM fungi. Whether a particular species was a root-associated EcM fungi was based on current literature [36]–[38]. The list of the MOTUs is given in Table S1. The table indicates the phylum and if the MOTUs is an EcM fungi.

Richness was used as diversity measure and was defined by the estimated number of MOTUs per sample. It was estimated, by rarefaction, at the lowest sequencing depths available, which were 1400 and 500 sequences per sample for the phyllosphere and the root-associated assemblages respectively. The rarefaction curves were computed for each sample (*i.e.* each plot and each habitat) with the *rarefy* function of the vegan R package [39]. Before rarefaction, one sequence per MOTU was randomly discarded to exclude the singletons from the dataset [42].To estimate the richness of a particular group *i*, such as the ascomycetes, the basidiomycetes, or the EcM fungi, we made the hypothesis that the ratio of MOTUs belonging to the group *i* within the unknown MOTUs (*i.e.* those for which the phylum was unknown, 10%, Table 2) was the same as within the assigned MOTUs. The estimated richness for the group *i* in sample *j* (R*_ij_*) was thus calculated as follows:
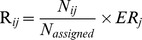



**Table 2:** Leaf and root samples description.

	Combined dataset	Leaf samples	Root samples
	(66 samples)	(33 samples*)	(33 samples*)
**Total number of 454 reads**	370 449	248 299	122 150
**Number of quality sequences**	228 104	149 946	78 158
**Range of sequences per sample**	-	1 506–15 830	552–3 622
**Range of sequences per elevation site**	-	5 691–30 380	4 716–10 040
**Number of MOTUs** 1	4 855	2 224	2 771
**Number of non singletons** 2	3 018	1 457	1 701
**Range of non singletons per sample**	-	134–487	134–342
**Range of non singletons per site**	-	255–634	375–568
**Number of nonsingletons assigned at the following taxonomic level**			
Phylum	1 725	967	813
Genus	525	143	415
Species	462	168	343
Unknown	306	179	130
**Number of nonsingletons assigned to the following phyla**			
Ascomycota	1 672 (55.4%)	958 (65.8%)	799 (47%)
Basidiomycota	935 (31.0%)	320 (22.0%)	667 (39.2%)
Zygomycota	97 (3.2%)	0	97 (5.7%)
Other or incertain fungal lineages	8 (0.3%)	0	8 (0.5%)
Unknown	306 (10.1%)	179 (12.3%)	130 (7.6%)
**Ecological mode**			
EcM^3^	306	0	306
Other or unknown	2 712	1 457	1 395

* Three plots ×11 elevation sites.

1 Molecular Operational Taxonomic Unit (97% similarity);

2 MOTUs supported by two sequences at least within the combined dataset;

3 including putative EcM fungi.

*N_ij_*, the number of MOTUs assigned to group *i* in sample *j*

*N_assigned_*, the number of MOTUs assigned at least to the phylum level in sample *j*

*ER_j_*, the estimated fungal richness of the sample *j*, estimated at the sequencing depth mentioned above.

The correspondence analysis (CoA) was used to discriminate the samples based on their species composition. The abundance matrix was rarefied at sequencing depth mentioned above with the *rrarefy* function of the vegan R package and then transformed into a presence/absence matrix. From this presence/absence matrix, the MOTUs detected in less than three plots were discarded to prevent distortion of the CoA by the rare MOTUs. The CoA was done with the *dudi.coa* function of the ade4 R package with default parameters [40].

The relationship between environmental variables was studied with a principal component analysis (PCA) and a Pearson's correlation table was used to select the variables that were not correlated for further analysis. The PCA was performed with the *PCA* function of the FactoMineR library. The Pearson's correlation coefficient and the p-value of the tests were obtained with the *cor.test* function of the stats library. To test for significant effects of selected environmental variables (mean annual temperature and soil pH) on fungal richnesses, mixed linear models were used with the *lme* function available from the nlme R package [41]. The interactions were not included in the models because they were not significant. To test for significant effects of the selected environmental variables on specific compositions, permutational analyses of variance (PERMANOVA) were performed with the *adonis* function available from the vegan R package. For each model, the elevation site was nested within the region factor (Alps, Vosges or Pyrenees). The permutations were restricted within the region using the strata option of the *adonis* function. The significance level was set at 0.05 for each test.

## Results

### Description of the dataset

We obtained 228 104 sequences that passed the quality filters. The mean number of sequences per sample following the cleaning steps was 4 544 and 2 368 for the leaf and root samples, respectively.

There were 6 475 MOTUs generated by the clustering step based on the sequence alignment. The second clustering based on the taxonomic assignation led to 4 855 MOTUs. The MOTUs supported by only one sequence within the entire dataset accounted for a high percentage (37.8%) and will be hereafter referred to as singletons. The number of singletons per sample was significantly correlated to the number of MOTUs per sample (F_1_,_64_ = 157.50, p-value<0.001, r^2^ = 0.707). The singletons were discarded, leaving 3 018 non-singleton MOTUs (hereafter MOTUs) for the analysis (Table 2).

The MOTUs were found in one (49.3%), two (28.8%) or three (21.9%) studied regions. Information on how many MOTUs were shared between and within regions is given in Table S2 and Figure S1). 29.0% were found in one out of the 11 sites and 1.5% were found in all the 11 elevation sites. A significant positive correlation was noted between the logarithm of the number of sequences per MOTUs and the frequency *i.e.* the number of plots in which the MOTUs were present (F_1,3016_ = 5721.0, p-value<0.001, r^2^ = 0.655).

The MOTUs were unassigned (10.1%), assigned at the phylum (57.2%), the genus (15.3%) or the species (17.4%) level only. Altogether, Dikarya represented 86.4% of the MOTUs and 96.1% of the phylum-assigned MOTUs (Table 2).

### Taxonomic diversity of leaf and root-associated fungal assemblages

We found a total of 1 457 nonsingletons MOTUs in the leaf samples and 1 701 MOTUs in the root samples (Table 2). These MOTUs represented a high diversity of fungal genera as 165 and 212 different fungal genera were detected in the phyllosphere and root-associated assemblages, respectively. The most diverse genera were *Cryptococcus* (25 MOTUs) and *Taphrina* (22 MOTUs) in the phyllosphere, and *Russula* (55 MOTUs) and *Cortinarius* (50 MOTUs) in the root samples. The phyllosphere harboured a higher proportion of ascomycetes compared to the root system (t_64_ = 18.96, p-value<0.001). There were 140 MOTUs shared by the leaf and root habitats but they were not equivalently represented in both habitats. In total, 306 MOTUs were classified as EcM fungi (Table 2) and belonged to 39 different genera. Among the EcM MOTUs, 289 (94.4%) were basidiomycetes.

### Environmental variations along the elevation gradients

According to the correlation table (Table S3), the environmental variables (mean annual temperature, annual rainfall, soil carbon content, soil nitrogen content, carbon∶nitrogen ratio, and soil phosphorus content) were all found to significantly correlate to elevation with the exception of soil pH. Moreover, with the exception of soil pH and C∶N ratio, all environmental variables were significantly related to each other. The soil pH was found to be only weakly related to the C∶N ratio. The C∶N ratio was also significantly related to the soil phosphorus content. The PCA confirmed these results. The two first axes of the PCA on environmental variables explained 49.3% and 20.2% of the variability, respectively (Figure S2). Nitrogen, phosphorus, temperature and rainfall were the main contributors to the first axis with approximately 20% each, whereas C∶N ratio and pH contributed to the second axis for 55% and 34% respectively. Therefore, only two variables, the mean annual temperature and the soil pH, were tested for explaining fungal assemblage diversity and composition (Table 3 and Table 4).

**Table 3:** Relationships between potential factors affecting leaf and root-associated fungal richness.

	Fixed variables		Random factors*		
	Mean annual temperature	Soil pH	Region	Site	Residual
Leaf MOTUs	−1.56 (0.162)	−1.17 (0.257)	25.43 (68.7)	5.08 (2.7)	16.39 (28.5)
Leaf Ascomycetes	−0.44 (0.674)	−0.61 (0.647)	12.40 (45.5)	6.09 (11.0)	12.12 (43.5)
Leaf Basidiomycetes	−1.88 (0.089)	−0.92 (0.366)	12.45 (66.7)	3.22 (4.5)	8.19 (28.9)
Root MOTUs	1.10 (0.309)	−0.64 (0.528)	0.003 (0)	14.78 (68.9)	9.94 (31.1)
Root Ascomycetes	**3.28 (0.014)**	0.61 (0.547)	2.11 (6.6)	5.92 (51.7)	5.32 (41.7)
Root Basidiomycetes	−0.32 (0.757)	0.01 (0.991)	0.001 (0)	7.78 (55.3)	7.00 (44.7)
Root EcM	0.20 (0.845)	−0.38 (0.706)	0.0006 (0)	5.16 (59.0)	4.30 (41.0)

The T-values (p-values) of the linear mixed model are presented for the variables tested and the standard deviations (% of the total variance explained [random+residual]) are presented for the random factors. Significant p-values in bold at 5%. The richness was estimated at 1 400 and 500 sequences per leaf and root samples respectively.

* Standard deviations associated with the region and site nested within region as random factors (% of the total random plus residual variance explained).

**Table 4:** Relationships between potential factors affecting leaf and root-associated fungal composition.

			Leaf		Roots	
Assemblage		Df	F (pvalue)	R2	F (pvalue)	R2
Fungi	region	2	9.768 (0.001)	0.263	4.903 (0.001)	0.174
	pH	1	3.867 (0.003)	0.052	5.109 (0.001)	0.091
	temperature	1	10.749 (0.001)	0.145	4.858 (0.001)	0.086
	region:site	7	2.744 (0.001)	0.258	2.238 (0.001)	0.278
	Residuals	21		0.282		0.372
Ascomycetes	region	2	10.470 (0.001)	0.274	5.326 (0.001)	0.188
	pH	1	4.064 (0.001)	0.053	5.394 (0.001)	0.095
	temperature	1	11.001 (0.001)	0.144	5.787 (0.001)	0.102
	region:site	7	2.765 (0.001)	0.253	1.994 (0.001)	0.246
	Residuals	21		0.275		0.370
Basidiomycetes	region	2	7.836 (0.001)	0.228	3.980 (0.001)	0.146
	pH	1	3.824 (0.002)	0.056	4.703 (0.001)	0.086
	temperature	1	10.496 (0.001)	0.153	3.933 (0.001)	0.072
	region:site	7	2.526 (0.001)	0.257	2.405 (0.001)	0.309
	Residuals	21		0.306		0.386
Ectomycorrhizal	region	2			3.724 (0.001)	0.140
fungi	pH	1			4.665 (0.001)	0.088
	temperature	1			4.247 (0.001)	0.080
	region:site	7			2.270 (0.001)	0.298
	Residuals	21				0.394

Permutational multivariate analysis of variance of the compositional dissimilarity.

### Variations in fungal richness along the elevation gradients

For leaf samples, the estimated richness ranged from 103 to 199 MOTUs per plot (81 to 154 ascomycetes and 12 to 55 basidiomycetes). For root samples, the estimated richness ranged from 88 to 157 MOTUs per plot (36 to 80 ascomycetes, 31 to 66 basidiomycetes, and 13 to 35 EcM MOTUs) (Table S4).

The richness was not significantly related to elevation (−0.78<t<2.08; 0.076<p-value<0.638; results not shown). The only exception was for the root ascomycete richness, which was significantly correlated to elevation (t = −3.47, p-value = 0.010) (Figure S3). However, there was a mid-elevational peak in species richness for root-associated fungi in the Pyrenees and in the Vosges (Figure S3).

**Figure 1:**
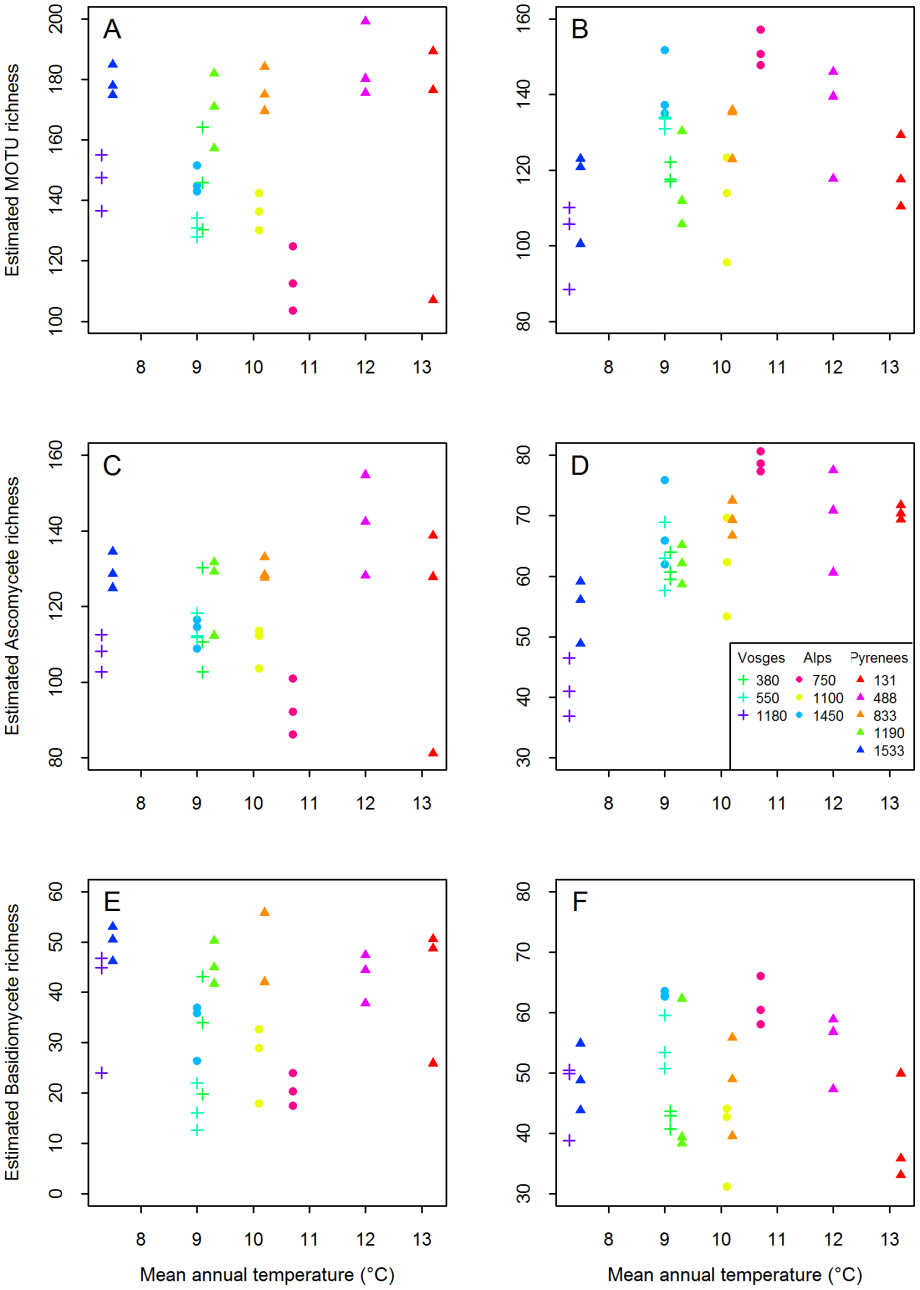
Relationships between fungal richness and mean annual temperature. Fungal richness from the phyllosphere (A, C, E) or associated with the fine-roots (B, D, F), either total richness (A, B), Ascomycetes richness (C, D) or Basidiomycete richness (E, F). The richness was estimated from rarefaction at a sequencing depth of 1 400 and 500 sequences per leaf and root samples, respectively. Samples corresponded to the Alps (closed dots), the Pyrenees (filled triangles) or the Vosges (plus sign) with different colours meaning different sites.

No relationship was found between richness and mean annual temperature except for the root ascomycete richness (t = 3.28, p-value = 0.014) (Figure 1; Table 3). Additionally, regardless of the subgroup considered (ascomycetes, basidiomycetes, EcM fungi), no significant relationship was found linking richness and other environmental variables (soil pH, C, N, C∶N, P, rainfall; data shown only for pH; Table 3). Similar conclusions were obtained when the diversity of fungal community was measured using the Shannon diversity index (data not shown).

The mountainous region (introduced as a random factor in the statistical analyses) was a notably different source of variability depending on the fungal assemblage. While leaf-associated fungal richness varied significantly depending on the region (45.5–68.7% of the total variance, Table 3, likelihood ratio of 6.6–7.8 with 0.005<p-value<0.060), root assemblage richness did not (likelihood ratio of about 10^−8^, p-value over 0.999, Table 3). For the global root-associated fungal community, most of the variation in richness was induced by the random site factor (likelihood ratio of 6.9–11.4 with p-value<0.010). The within-site variation between the 3 plots of 5 trees represented between 28 and 44% of the total variation depending on the assemblage considered (Table 3).

### Variations in fungal composition along the elevation gradients

**Figure 2:**
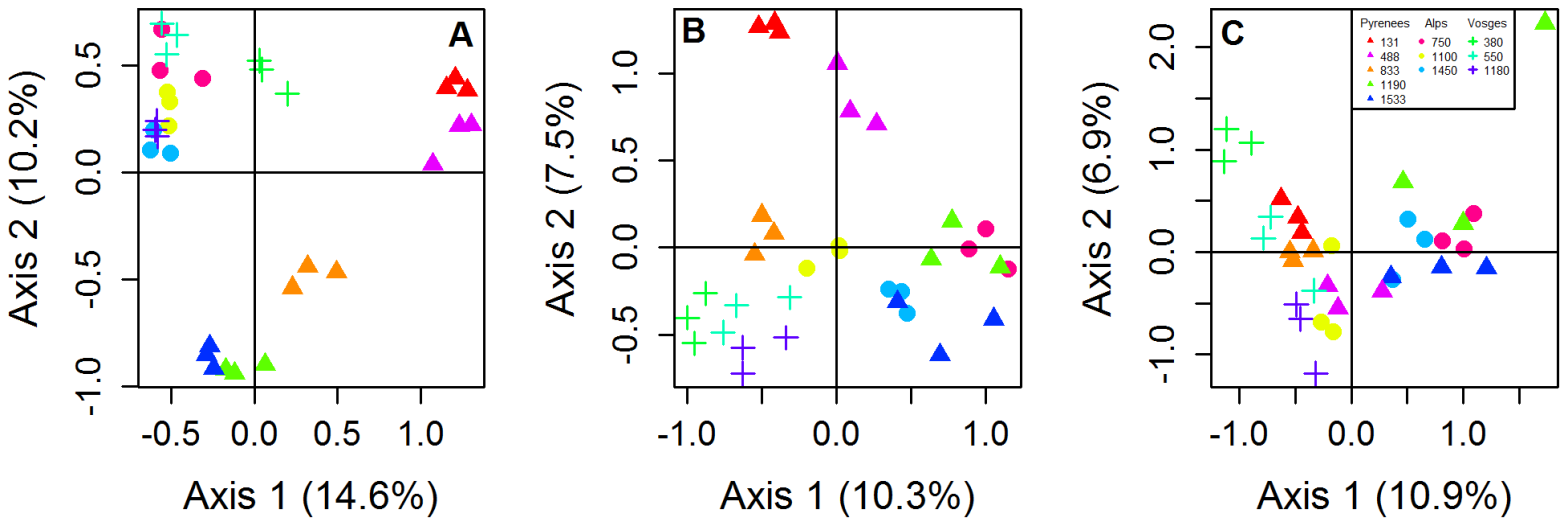
Correspondence analysis representing the distribution of the samples according to the presence/absence of fungal Molecular Operational Taxonomic Units (MOTUs). There were 512 phyllosphere-associated MOTUs (A), 472 root-associated MOTUs (B), and 120 root-associated EcM MOTUs (C). Samples were from the Alps (closed dots), the Pyrenees (filled triangles) or the Vosges (plus sign) with different colours meaning different sites. Percentage of variance into brackets.

The CoA showed that relatively little variation in the species composition existed between the 3 plots within a site (Figures 2A and 2B). Still, the within-site variability can be measured by the residuals of the permutational multivariate analysis of variance and represented about 28–30% for leaf-associated communities and slightly more, 37–39% for root-associated communities (Table 4).

The phyllosphere fungal assemblage of the Pyrenees was very different from those of the Alps and the Vosges, which were quite similar to each other (Figure 2A). The region effect represented about 25% of the variability in the PERMANOVA (Table 4). The sites were distributed on the first and second axes according to the elevation, in particular for the Pyrenees, with low elevation sites having high values on the first axis and low values on the second axis (Figure 2A). This distribution indicates that the assemblage in the high elevation sites may be different from the low elevation sites. The ten leaf MOTUs that contributed the most on the first axis of the CoA contributed for over 0.86% each. The permutational multivariate analysis of variance confirmed that temperature was the main environmental factor affecting the composition of leaf-associated fungal communities (Table 4, see also [11]). Mean site temperature represented about 15% of the variation of the leaf-associated communities compared to about 5% for soil pH.

Concerning the root assemblage, the regional effect accounted for 15% of the variability (Table 4). On Figure 2B which relates to root-associated fungi, the three plots from the two sites in the Pyrenees which had strong positive values on the second axis were the two lowest elevation sites from the Pyrenees region (Lourdes and Laveyron, Table 1). The permutational multivariate analysis of variance showed that soil pH and site mean temperature explained similar levels of the root-associated communities variability, whether the entire community was considered or only part of it (ascomycetes, basidiomycetes or EcM species). Site temperature explained about 8% of the variability while soil pH explained about 9% (Table 4). Some EcM species were only detected in the warmer sites (≥12.0°C) *e.g. Russula solaris* and *Russula ingwa*. On the contrary, *Melanogaster* sp2 and *Clavulina amethystina* were only detected in the colder sites (≤7.5°C). Twenty-three *Russula* spp, 16 *Cortinarius* spp, 14 *Hypochrea* spp, 10 *Cryptococcus* spp., eigth *Lactarius* spp., five *Boletus* spp., and other species from other genera were not found in sites with a soil pH>5.

The EcM fungi belonged to 39 different genera. From 18 to 28 were detected in a single site. Twenty-three of the observed EcM genera were detected in the three regions, 10 were in 2 regions and 6 only in one region. Nine genera were detected in all of the 11 elevation sites.

## Discussion

### Fungal richness does not decline along elevation gradients

Our results show that no clear relationship exists between fungal richness and elevation across a broad range (from 131 to 1533 m) (Figure S3). This finding adds to studies that have reported no patterns or inconclusive patterns of fungal richness along elevation gradients [17]–[18], [22] but differs to previous studies which found lower richness levels in high-elevation sites compared to low or mid-elevation sites [12], [19]. A possible explanation for these differences is the relative homogeneity of host factor. For instance, the diversity of EcM hosts is known to actively influence the diversity of EcM assemblage [25], [43]. This factor was controlled along the three elevation gradients in our study (dominated beech forest), whereas it was not controlled in several previous studies [12], [19]. A poor estimate of the diversity of microbial community could also impede the detection of a clear pattern of richness along the gradients. However, similar conclusions are obtained using diversity index such as the Shannon index suggested in the literature [44].

In our study, the fungal richness does not correlate with the climatic and soil variables that covaried with elevation (mean annual temperature, annual rainfall, soil carbon content, soil nitrogen content, carbon∶nitrogen ratio, and soil phosphorus content) (Table 3). Our results are not in agreement with previous studies reporting that the mean annual temperature and precipitation drive EcM species richness along elevation gradients [12], [16]. Interestingly, only ascomycete species richness peaked at mid-temperature (Figure 1D). This richness distribution is significantly validated by a quadratic curve illustrating a mid-domain effect model (data not shown). Previously, different analyses of elevational diversity revealed patterns of mid-elevational peaks in mammal species richness [45]–[46], illustrating this mid-domain effect [47]. Because this trend is not observed for basidiomycetes, it is possible that the richness of ascomycetes and the richness of basidiomycetes are not related to the same environmental variables. This finding suggests that considering the whole fungal assemblage might blur the relationship of sub-assemblage richness with environmental gradients. A confirmation of this finding is needed through additional studies. That is why we recommend studying the ascomycetes and the basidiomycetes independently.

Furthermore, we observed that the fungal richness does not correlate to the soil pH (Table 3). This finding corroborates a similar observation of a non-significant relationship between the soil pH and fungal richness [24].

### The composition of leaf-associated fungal assemblages covaries with temperature

Our results confirm that mean annual temperature could be of main importance in structuring the phyllosphere fungal assemblages at large geographic scales [11] (Table 4). Only one sampling in summertime has been done to avoid taking the seasonal variability into account [11]. Care should be taken about the conclusions as factors such as total rainfall or soil phosphorus content were strongly correlated to the temperature and thus, the exact causal factor could not be determined. Some recent studies showed that the leaf-associated fungal assemblages are spatially structured, from the regional scale [9] to the single tree canopy scale [48]–[49] and along elevation gradients [50]. Although these fungi are often generalists with a cosmopolitan distribution, these assemblages are structured by both abiotic factors such as the mean annual temperature [11] or rainfall [50], and biotic factors such as the host genotype [49]. The difference in fungal assemblages between, in one hand, the Pyrenees and, in another hand, the Alps and the Vosges could be the result of a higher initial sequencing effort in the Pyrenees although all the assemblages were randomly downsampled at a similar sequencing depth.

### The composition of root-associated fungal assemblages covaries both with soil pH and temperature

Several recent studies showed that the belowground fungal composition varies along elevation gradients [12], [19], [20], [22]. In this study, the composition of root-associated fungi first correlat with the soil pH and secondly with the temperature (Table 4). However, the soil pH correlated with the C∶N ratio and because the mean annual temperature correlated with two other soil variables (nitrogen and phosphorus) the direct effect of climate cannot be ascertained (Figure S2 and Table S3). Nevertheless, the soil characteristics were previously reported as drivers of the microbial assemblage. Indeed, our results show that fungal composition is strongly related to soil pH confirming previous reports on soil fungal and bacterial communities [24], [51].

Our results suggest that temperature might be an important factor in shaping EcM specific composition [12], [16] although it was not possible to distinguish the effect of the climatic variables and the correlated soil variables. The climatic variables could drive EcM composition indirectly by the effect of climate on vegetation through root status and turnover for example. Indeed, it is known that a major structuring factor of EcM assemblages is the host family [53]. The beech-dominated stands were explicitly chosen to limit this biotic effect. This may explain why the region effect (Alps, Pyrenees, Vosges) is of less importance for explaining root-associated basidiomycetes assemblage diversity as EcM fungi closely associated with their beech host represent a large part of this assemblage. Some EcM fungi show a high presence throughout the study sites such as *Cenococcum spp.* Its ubiquity within the French beech forest has already been reported and could result from the pooling of different cryptic species as it is now recognized that *C. geophilum* is a species complex [52], each species possibly having a particular niche. For instance, the MOTU *Cenococcum sp1* was restricted to plots where the pH was above 5.

## Conclusions

According to our results, the above-ground and below-ground fungal assemblages do not follow similar environmental drivers. The phyllosphere assemblage was found to be dominated by ascomycetes, as has already been described [9], [54] whereas both ascomycetes and basidiomycetes contributed to root-associated fungal assemblage in a similar proportion, even if EcM fungi are dominated by basidiomycetes. While the phyllosphere assemblage appeared to be largely related to climatic variables, the root-associated assemblage was related to both edaphic and climatic variables. To go further, it appears important to analyse the data at lower taxonomic levels (*e.g.*, [24]) or taking into account the ecological trait differences. It is possible that the fungal taxonomic groups are too heterogeneous to be pooled into one assemblage. Considering the whole assemblage might therefore blur the relationship of sub-assemblage with environmental gradients and impede our understanding of fungal community ecology.

## Supporting Information

Figure S1 **Venn diagrams with the number of non singleton MOTUs.** MOTUs were found in the different regions (A), from the phyllosphere at the different elevation sites (B) and from the root-associated assemblages at the different elevation sites (C).
(PPT)

Figure S2 **Principal component analysis of the environmental variables.** Note that the annual precipitation and elevation are overlapped.
(PDF)

Figure S3 **Relationships between fungal richness and elevation.** Fungal richness from the phyllosphere (A, C, E) or associated with the fine-roots (B, D, F), either total richness (A, B), Ascomycetes richness (C, D) or Basidiomycete richness (E, F). The richness was estimated from rarefaction at a sequencing depth of 1 400 and 500 sequences per leaf and root samples, respectively. Samples corresponded to the Alps (closed dots), the Pyrenees (filled triangles) or the Vosges (plus sign) with different colours meaning different sites.
(TIF)

Table S1 **List of the MOTUs with the taxonomic assignation and classification as ectomycorrhizal fungi** (if applicable).
(XLS)

Table S2 **Percentage of non-singleton MOTUs shared or not shared between the elevation sites of each region.**
(PPTX)

Table S3 **Pearson coefficients of correlation between the environmental variables.** Significant correlations are indicated with asterisks for the following thresholds: ***<0.001<**<0.01<*<0.05.
(XLS)

Table S4 **Estimated richness per plot.** The richness was estimated from rarefaction at a sequencing depth of 1 400 and 500 sequences per leaf and root samples, respectively. See Materials and Methods for the detailed estimation methods.
(XLS)
