# Supplementary material for: Leaf and Root-Associated Fungal Assemblages Do Not Follow Similar Elevational Diversity Patterns
Source: PLoS One. 2014 Jun 27;9(6):e100668. doi: 10.1371/journal.pone.0100668 (PMC4074112; doi:10.1371/journal.pone.0100668)
Supplement: Figure S1 — Venn diagrams with the number of non singleton MOTUs. MOTUs were found in the different regions (A), from the phyllosphere at the different elevation sites (B) and from the root-associated assemblages at the different elevation sites (C). (PPT) [file pone.0100668.s001.ppt]

## Slide 1
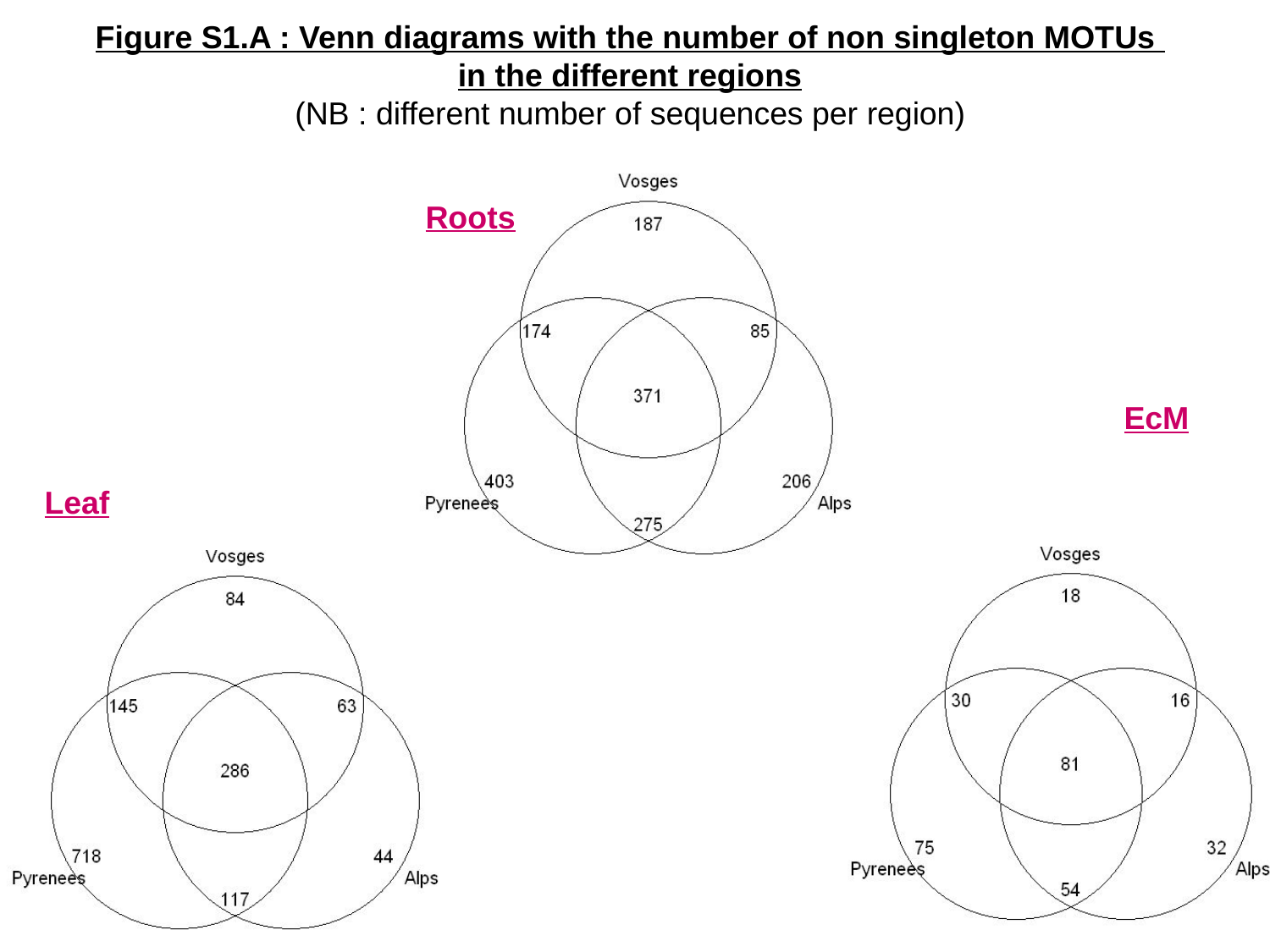

Figure S1.A : Venn diagrams with the number of non singleton MOTUs
in the different regions
(NB : different number of sequences per region)
Roots
EcM
Leaf

## Slide 2
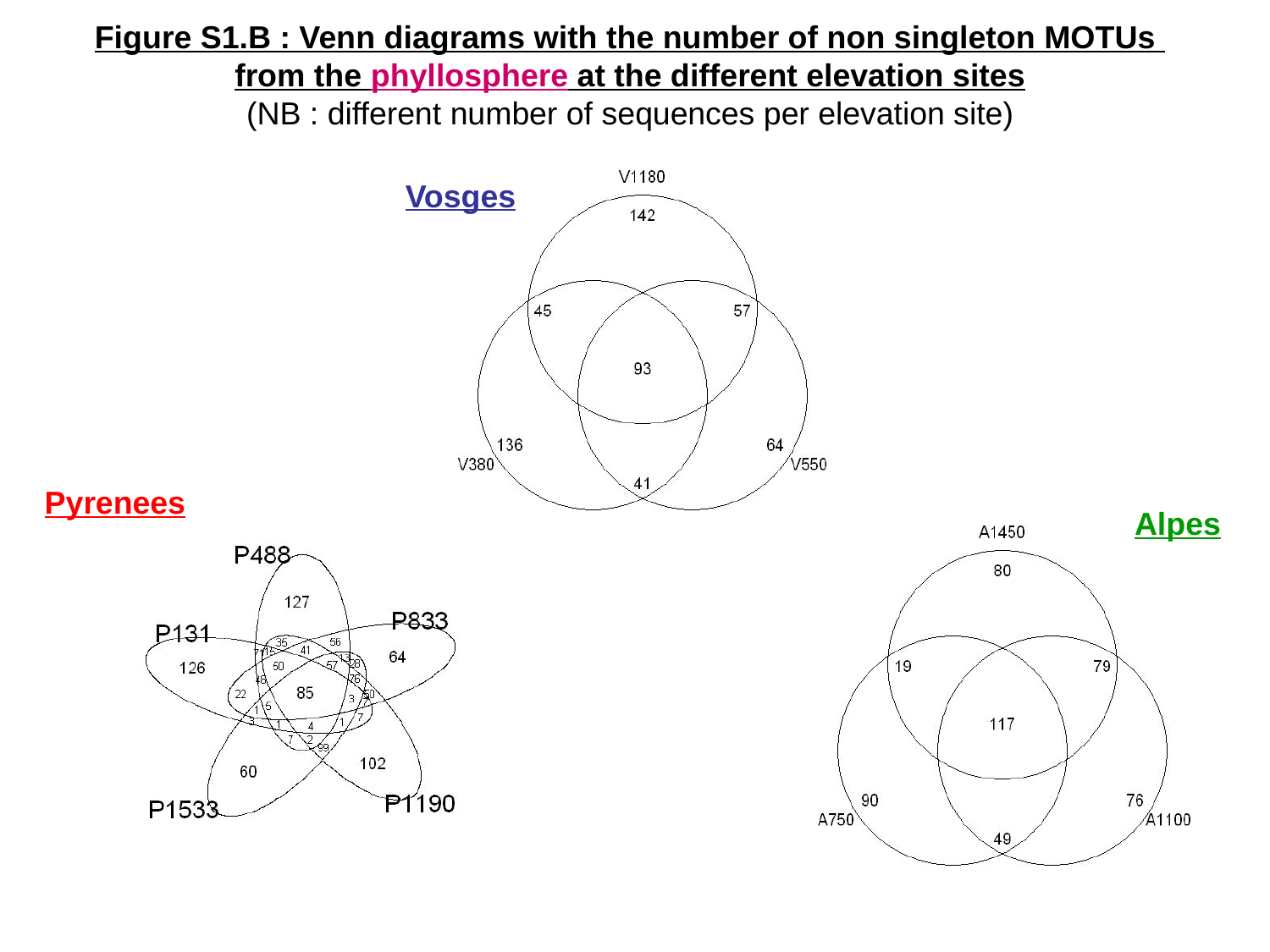

Figure S1.B : Venn diagrams with the number of non singleton MOTUs
from the phyllosphere at the different elevation sites
(NB : different number of sequences per elevation site)
Vosges
Pyrenees
Alpes

## Slide 3
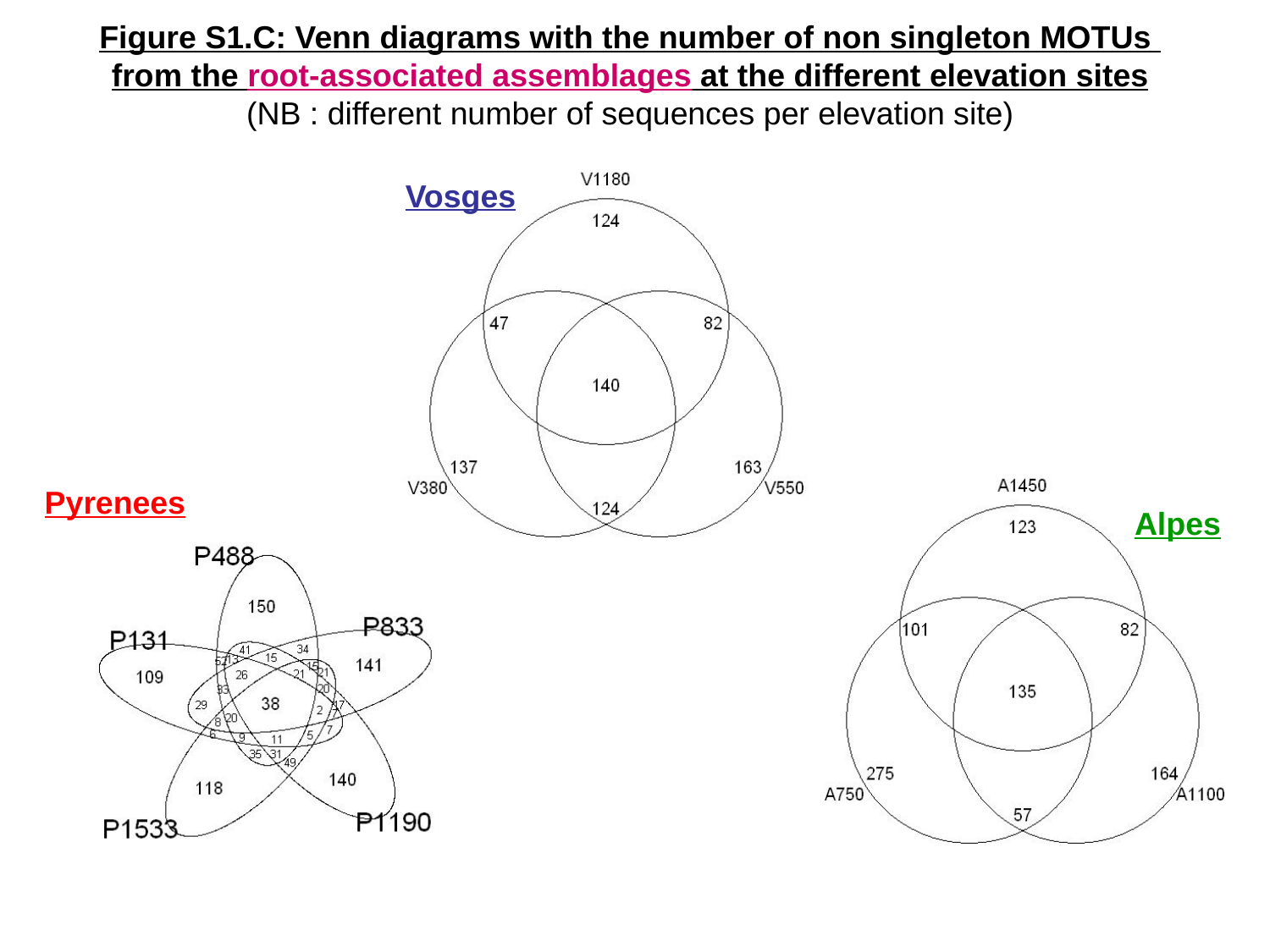

Figure S1.C: Venn diagrams with the number of non singleton MOTUs
from the root-associated assemblages at the different elevation sites
(NB : different number of sequences per elevation site)
Vosges
Pyrenees
Alpes
