# Supplementary figures and images for: Leaf and Root-Associated Fungal Assemblages Do Not Follow Similar Elevational Diversity Patterns
Source: PLoS One. 2014 Jun 27;9(6):e100668. doi: 10.1371/journal.pone.0100668 (PMC4074112; doi:10.1371/journal.pone.0100668)

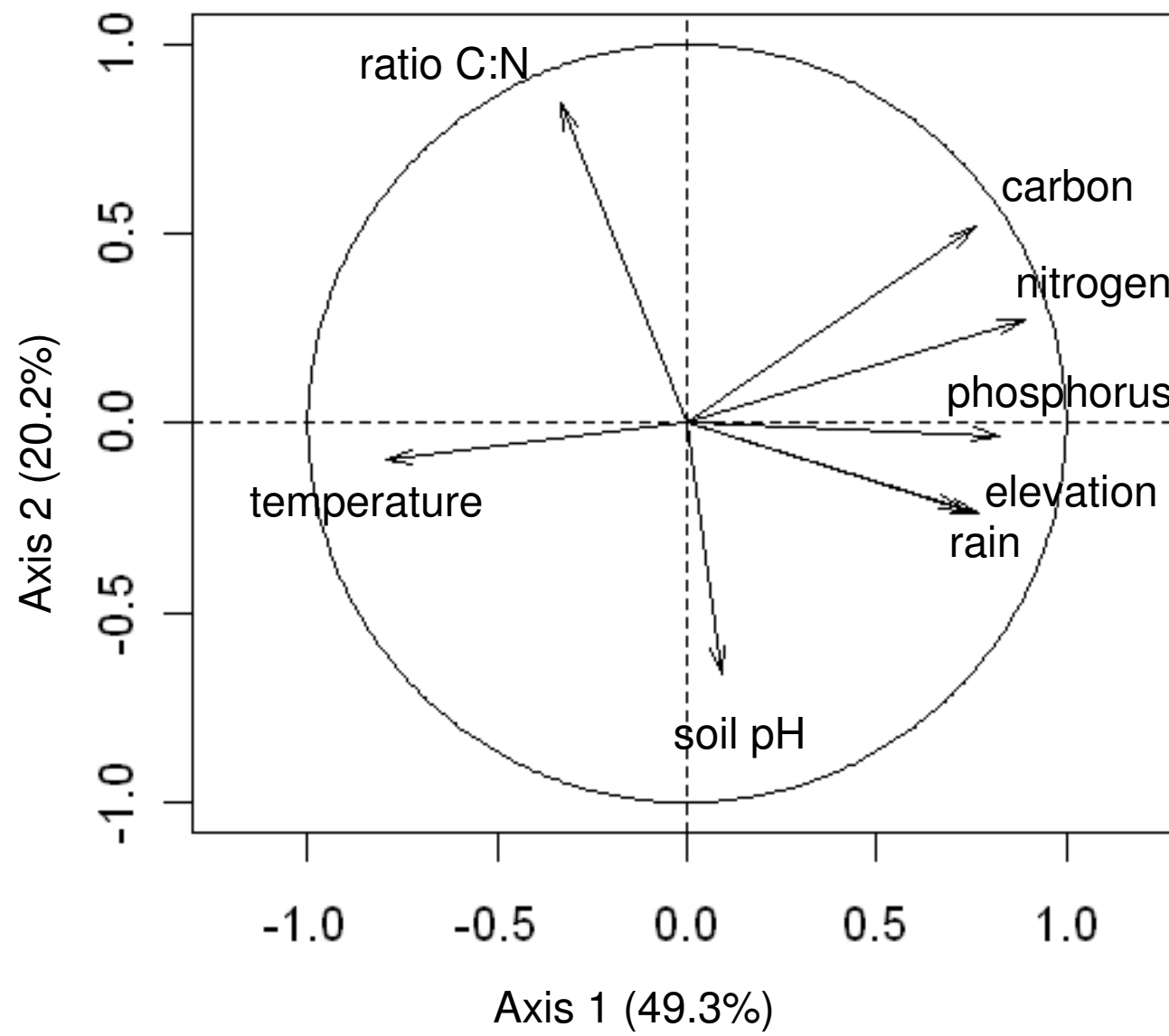

Supplement: Figure S2 — Principal component analysis of the environmental variables. Note that the annual precipitation and elevation are overlapped. (PDF) [file pone.0100668.s002.pdf]

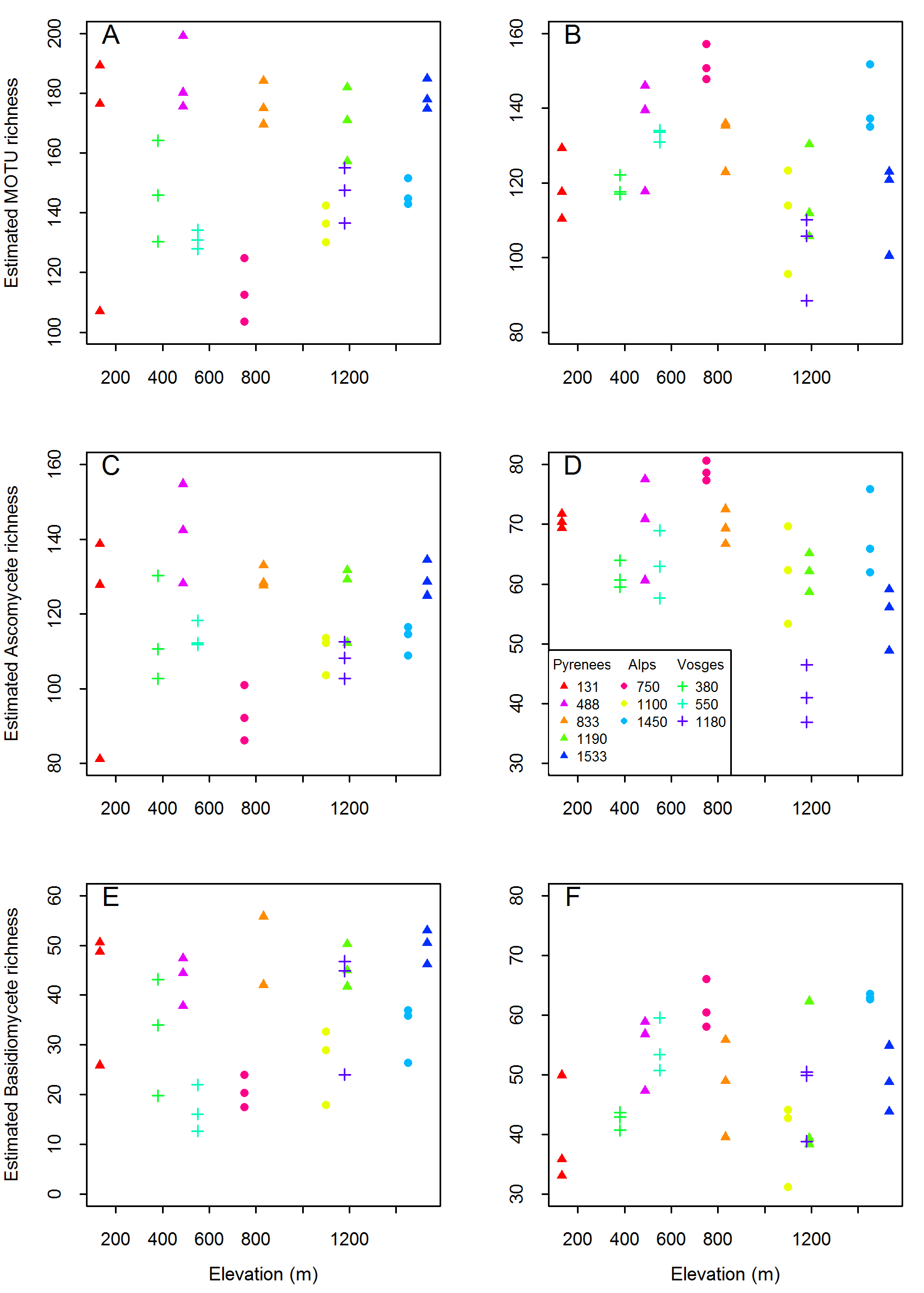

Supplement: Figure S3 — Relationships between fungal richness and elevation. Fungal richness from the phyllosphere (A, C, E) or associated with the fine-roots (B, D, F), either total richness (A, B), Ascomycetes richness (C, D) or Basidiomycete richness (E, F). The richness was estimated from rarefaction at a sequencing depth of 1 400 and 500 sequences per leaf and root samples, respectively. Samples corresponded to the Alps (closed dots), the Pyrenees (filled triangles) or the Vosges (plus sign) with different colours meaning different sites. (TIF) [file pone.0100668.s003.tif]
